# Baseline high-resolution maps of organic carbon content in Australian soils

**DOI:** 10.1038/s41597-023-02056-8

**Published:** 2023-03-31

**Authors:** Alexandre M. J-C. Wadoux, Mercedes Román Dobarco, Brendan Malone, Budiman Minasny, Alex B. McBratney, Ross Searle

**Affiliations:** 1 Sydney Institute of Agriculture & School of Life and Environmental Sciences, The University of Sydney, Camperdown, Australia; 2 CSIRO Agriculture and Food, Black Mountain, ACT Australia; 3 CSIRO Agriculture and Food, 306 Carmody Road, St Lucia, QLD Australia

**Keywords:** Environmental sciences, Ecology

## Abstract

We introduce a new dataset of high-resolution gridded total soil organic carbon content data produced at 30 m × 30 m and 90 m × 90 m resolutions across Australia. For each product resolution, the dataset consists of six maps of soil organic carbon content along with an estimate of the uncertainty represented by the 90% prediction interval. Soil organic carbon maps were produced up to a depth of 200 cm, for six intervals: 0–5 cm, 5–15 cm, 15–30 cm, 30–60 cm, 60–100 cm and 100–200 cm. The maps were obtained through interpolation of 90,025 depth-harmonized organic carbon measurements using quantile regression forest and a large set of environmental covariates. Validation with 10-fold cross-validation showed that all six maps had relatively small errors and that prediction uncertainty was adequately estimated. The soil carbon maps provide a new baseline from which change in future carbon stocks can be monitored and the influence of climate change, land management, and greenhouse gas offset can be assessed.

## Background & Summary

In the last two decades, there has been a growing interest in estimating soil organic carbon (SOC) content and stocks for management (e.g. carbon sequestration), economic (e.g. greenhouse gas emission trading schemes, commercial incentives for Net Zero targets)^1^ and scientific (e.g. dynamic of carbon cycle) purposes. Soils are an essential component of the ecosystem carbon cycle, storing approximately two-thirds of the total terrestrial organic carbon pool^2^. Organic carbon is also a key indicator of the overall soil functioning. It is the main constituent of soil organic matter and is related to most soil functions such as water and nutrient cycling, the productivity of plants, carbon storage and climate mitigation, among others^3^.

The total SOC concentration is conventionally measured at a point using laboratory techniques (e.g. the Walkley-Black method^4^ or high-temperature combustion), the values of which can be used in models of soil C dynamics models such as RothC^5^). Organic carbon, however, is a continuum of compounds with different origins, multiple stages of decomposition and decay and chemical composition^6^. The processes that control organic carbon composition vary spatially and with depth, depending on soil, climate, land use and management practices and are controlled by a myriad of biotic and abiotic factors^7^. Several recent lines of work have therefore been developed to model the spatial distribution of SOC at regional^8^, national^9^ and continental^10^ scales. The mapping of SOC also reflects an increasing demand for spatially explicit organic carbon assessment to be used. For example, to prioritize local actions in soil carbon sequestration and to monitor SOC change over time. SOC maps are also used as input into Earth System Models and are relevant to calibrate and initiate mechanistic simulation models of the terrestrial carbon cycle^11^.

SOC maps are usually made using statistical or non-statistical models that exploit the quantitative relationships between point-measured values of SOC and a set of environmental covariates that control SOC spatial distribution^11,12^. Various methods can be used for this purpose, including geostatistical methods that rely on the variogram and kriging and recently more complex algorithmic tools from machine learning^13^. These models are used to predict the SOC at unobserved locations using the fitted relationship obtained at observation points and the spatially explicit covariates, such as terrain attributes and remote sensing imagery.

In Australia, digital maps of SOC have been produced extensively for nearly two decades. The Soil and Landscape Grid of Australia (SLGA)^14^, for example, used a large soil inventory composed of more than 27,000 sites where SOC is recorded, either measured or inferred with spectroscopic techniques. These SOC maps have been extensively used for national carbon accounting and to monitor soil carbon change. However, data collection and processing techniques have progressed rapidly in the last 8 years. It is worthwhile to include these recent developments to produce new high-resolution maps of SOC in Australia.

In this paper, we present updated continental-scale maps of SOC. These maps are part of the new version of the SLGA and will be the new baseline maps of Australia from which carbon stock can be estimated and changes in SOC can be monitored. The resolution at which the maps are produced (90 m × 90 m and 30 m × 30 m) enables applications from regional assessment to local-scale soil management. The maps are produced for six depth intervals, following the specifications of the GlobalSoilMap project^15^ and we also produced maps of the prediction uncertainty. The maps are based on measured total organic carbon (TOC) content in soils compiled from various sources which represent the most comprehensive dataset on SOC currently available in Australia.

## Methods

### Organic carbon data

Data on total organic carbon (TOC) concentration (%) was extracted with the Soil Data Federator developed by CSIRO with support from the Terrestrial Ecosystem Research Network (TERN). The Soil Data Federator is a web API that compiles soil data from different institutions and government agencies throughout Australia. The SOC data used in this study are publicly available through the Soil Data Federator (https://esoil.io/TERNLandscapes/Public/Pages/SoilDataFederator/SoilDataFederatorHelp.html) managed by CSIRO^16^. The laboratory methods for total organic carbon included in the study are presented in Table 1. We selected TOC data from the period 1970–2020 to get a compromise between the representativity of current TOC concentration and spatial coverage. The data was error checked and processed to harmonize units, excluding duplicates and potentially wrong data entries (e.g. missing upper or lower horizon depths, extreme TOC values, unknown sampling date). Additional TOC measurements from the Biome of Australian Soil Environments (BASE) contextual data^17^ were also included in the analyses. TOC concentration for BASE samples was determined by the Walkley-Black method^4^ (method 6A1 in Table 1). Upper limits for TOC concentration by biome and land cover classes were set according to published literature, and consistent datasets (Australian national Soil Carbon Research Program (SCaRP) and BASE), see refs. ^17,18^ and data exploration to exclude unrealistic TOC values (e.g. maximum TOC = 30% in temperate forests, maximum TOC = 14% in temperate rainfed pasture). Since TOC concentration in Australian ecosystems has been underestimated by previous SOC maps^19^, we did not set conservative TOC upper limits, knowing that the machine learning model would likely underestimate high SOC values.

**Table 1:** Datasets used for spatial modelling along with the laboratory method and reference.

Laboratory methods	Description	Reference
6A1, 6A1_UC	Organic C. Walkley and Black wet Oxidation by dichromate-sulfuric acid. TOC approximate due to incomplete chemical reaction	^4^
6B2	Total C. Dumas high-temperature combustion, volumetric CO2 measurement (no soil pretreatment). Measures TOC in absence of carbonates/bicarbonates	^32^
6B2b	Total organic C. Dumas high-temperature oxidative combustion, infrared/thermal conductivity detection (no soil pretreatment).	^32^
6B3, 6B3a	Total organic C. Dumas high-temperature oxidative combustion furnace, infrared/thermal conductivity detection. The results are corrected for the percentage of cabonate/bicarbonate determined separately.	^33^

**Fig. 1:**
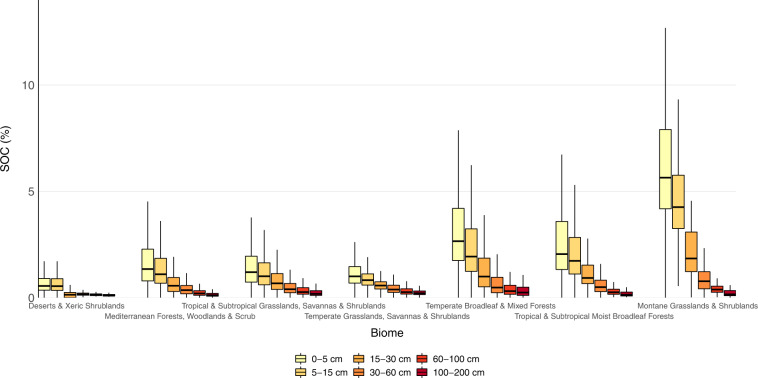
Values of total organic carbon concentration by biome and land cover after data cleaning and depth standardization.

Data for TOC concentration come from bulk soil samples taken at various depth intervals in the soil profile. To standardize the depth intervals, we built a mass-preserving depth function using the equal-area quadratic spline. The mathematical description of this function and its application to build continuous depth intervals over the soil profile have been extensively described in the literature^20,21^. The equal-area quadratic spline function was fitted to the whole collection of pre-processed TOC data, and then values extracted for the 0–5 cm, 5–15 cm, 15–30 cm, 30–60 cm, 60–100 cm, and 100–200 cm depth intervals, following GlobalSoilMap specifications^15^. Boxplots with TOC values by biome and land cover after data cleaning and depth standardization are shown in Fig. 1. A total of 90,025 measurements of TOC are used hereafter for mapping.

### Environmental covariates

We collected a set of 57 spatially exhaustive environmental covariates made available by the Terrestrial Ecosystems Research Network (TERN), covering Australia and representing proxies for factors influencing SOC formation and spatial distribution: soil properties, climate, organisms/vegetation, relief and parent material/age. The covariates were reprojected to the WGS84 (EPSG:4326) projection and cropped to the same spatial extent. All covariates were resampled using bilinear interpolation or aggregated to conform with a spatial resolution with a grid cell of 90 m × 90 m and 30 m × 30 m. The list of covariates along with their unit and reference is provided in Table 2. The covariates used in this study are freely available through the link https://data.tern.org.au/landscapes/slga/NationalMaps/SoilAndLandscapeGrid/. Instructions for accessing the covariate rasters as Cloud Optimised GeoTiffs are provided at https://esoil.io/TERNLandscapes/Public/Pages/SLGA/GetData-COGSDataStore.html.

**Table 2:** List of environmental covariates with unit and associated reference when applicable.

Factor	Predictor variable	Unit	Reference
Soil	Depth-specific soil clay content	percent	^34^
	Depth-specific soil sand content	percent	^34^
Climate	Mean annual aridity index (annual precipitation/annual potential evaporation)	index	^35^
	Annual potential evaporation	mm	^35^
	Minimum monthly potential evaporation	mm	^35^
	Maximum monthly potential evaporation	mm	^35^
	Prescott Index generated by using Prescott index = 0.445 P/E0.75	index	—
	Annual precipitation	mm	^35^
	Minimum monthly precipitation	mm	^35^
	Precipitation seasonality 1- solstice seasonality composite factor ratio	ratio	^35^
	Precipitation seasonality 2- equinox seasonality composite factor ratio	ratio	^35^
	Maximum monthly precipitation	mm	^35^
	Short-wave solar radiation - annual mean (SRAD data)	MJ/m2/day	^35^
	Minimum temperature – Annual mean	°C	^35^
	Annual temperature range	°C	^35^
	Maximum temperature - Annual mean	°C	^35^
	Annual atmospheric water deficit (annual precipitation – annual potential evaporation)	mm	^35^
Organisms/vegetation	Long-term average NDVI Q1	unitless	
	Long-term average NDVI Q2	unitless	
	Long-term average NDVI Q3	unitless	
	Long-term average NDVI Q4	unitless	
	Landsat Fractional cover - Bare Soil -Maximum of the timeseries - 1987–2019	percent	^36^
	Landsat Fractional cover - Non Photosynthetic Vegetation - Maximum of the timeseries - 1987–2019	percent	^36^
	Landsat Fractional cover - Photosynthetic Vegetation - Maximum of the timeseries - 1987–2019	percent	^36^
	Landsat Fractional cover - Bare Soil - Mean of the timeseries - 1987–2019	percent	^36^
	Landsat Fractional cover - Non Photosynthetic Vegetation - Mean of the timeseries - 1987–2019	percent	^36^
	Landsat Fractional cover - Photosynthetic Vegetation - Mean of the timeseries - 1987–2019	percent	^36^
	Landsat Fractional cover - Bare Soil Minimum of the timeseries - 1987–2019	percent	^36^
	Landsat Fractional cover - Non Photosynthetic Vegetation - Minimum of the timeseries - 1987–2019	percent	^36^
	Landsat Fractional cover - Photosynthetic Vegetation - Minimum of the timeseries - 1987–2019	percent	^36^
	Landsat Fractional cover - Bare Soil - Standard deviation of the timeseries - 1987–2019	percent	^36^
	Landsat Fractional cover - Non Photosynthetic Vegetation - Standard deviation of the timeseries - 1987–2019	percent	^36^
	Landsat Fractional cover - Bare Soil - Standard deviation of the timeseries -1987–2019	percent	^36^
	Fraction of Photosynthetically Active Radiation (FPAR) - AVHRR - Maximum Value in Timeseries	percent	^37^
	Fraction of Photosynthetically Active Radiation (FPAR) - AVHRR - Mean Value in Timeseries	percent	^37^
	Fraction of Photosynthetically Active Radiation (FPAR) - AVHRR - Median Value in Timeseries	percent	^37^
	Fraction of Photosynthetically Active Radiation (FPAR) - AVHRR - Minimum Value in Timeseries	percent	^37^
	National Dynamic Land Cover Dataset Mean of the timeseries 2000 - 2008	unitless	^38^
	Landsat 2000–2010 Persistent Green-Vegetation Fraction	unitless	^39^
Relief	Elevation 3 Second - Shuttle Radar Topography Mission	metre	^40^
	Multi-resolution Ridgetop Flatness	unitless	^41^
	Multiresolution Index of Valley Bottom Flatness (MRVBF)	unitless	^42^
	Plan curvature	unitless	^43^
	Profile curvature	unitless	^43^
	relief roughness	unitless	^43^
	Slope	percent	^44^
	Topographic wetness index	unitless	^43^
Parent material/age	Total Magnetic Intensity (TMI) Gravity Grid of Australia	unitless	^45^
	Radiometric grid of Australia (Radmap) v4 2019 - Filtered dose	unitless	^46^
	Radiometric grid of Australia (Radmap) v4 2019 - Potassium	percent	^46^
	Radiometric grid of Australia (Radmap) v4 2019 - Thorium	ppm	^46^
	Radiometric grid of Australia (Radmap) v4 2019 - Uranium	ppm	^46^
	Radiometric grid of Australia (Radmap) v4 2019 - Thorium Potassium ratio	ratio	^46^
	Radiometric grid of Australia (Radmap) v4 2019 - Uranium Thorium ratio	ratio	^46^
	Radiometric grid of Australia (Radmap) v4 2019 - Uranium Potassium ratio	ratio	^46^
	Radiometric grid of Australia (Radmap) v4 2019 - Uranium Thorium ratio	ratio	^46^
	Weathering index	unitless	^47^

All covariates are in geographic coordinates with 3 arc second grid cell (about 90 m) or 1 arc second grid cell (about 30 m) resolution with coordinate system WGS84 (EPSG:4326) and extent: 112.99958°E - 153.99958°E; 10.0004°S - 44.00042°S.

### Mapping

The spatial distribution of soil TOC concentration is driven by the combined influence of climate, vegetation, relief and parent materials^22^. We thus modelled TOC concentration as a function of environmental covariates representing biotic and abiotic control of TOC. The measurements of SOC and their corresponding values of environmental covariates from Table 2 at the same measurement locations were used to fit the mapping model. For the mapping, we used a machine learning algorithm called quantile regression forest.

#### Quantile regression forest

Quantile regression forest^23^ is an ensemble of decision trees. A decision tree is built by partitioning the covariate dataset from the calibration dataset. A number of partitions are evaluated and a splitting metric, the variance, is used to evaluate the partitions. The partition with the smallest splitting metric is selected and undergoes the same procedure until a user-defined parameter, the minimum node size, is reached. For a single tree, the prediction is taken as the average prediction of the values at the end of the node of the tree.

The decision tree is extended by the process of bagging (i.e. bootstrap and aggregating)^24^, which aims to build an ensemble of decision trees called a random forest. In a random forest, a large number of decision trees is built on bootstrap samples of the original calibration data. For each tree, a random perturbation (i.e. bagging) is introduced during partition where only a subset of size mtry from the original number of covariates in the calibration data is used for partition. The final prediction from the random forest is simply the aggregation through averaging of all the decision tree predictions. Extending the standard random forest to QRF is straightforward. Instead of obtaining a single statistic, that is the mean prediction from the decision trees in the random forest, we report all the target values of the leaf node of the decision trees. With QRF, the prediction is thus not a single value but a cumulative distribution of the TOC prediction at each location, which can be used to compute empirical quantile estimates.

Fitting a QRF model is thus based on three user-defined parameters: the partition subset size mtry, the number of trees ntree and the stopping criterion for the tree splitting nodesize. We fitted a QRF model for each depth interval with parameters mtry and nodesize held to their default values. mtry is rounded down to the square root of the total number of covariates and nodesize was set to 5. To compromise between computational load and accuracy we fixed the number of trees to 250. We tested parameter tuning using a random grid-search procedure for the three QRF parameters, using 1000 parameter set combinations and the mean of the square error as criterion obtained from a 10-fold cross-validation strategy. We found that parameter tuning was very computationally demanding for a negligible improvement in prediction accuracy. Thus, we did not proceed any further with QRF parameter tuning. We used the R programming language and the ranger^25^ package for model fitting and prediction.

#### Evaluation of the prediction and uncertainty quantification

##### Model prediction

Each depth-specific model of TOC was validated based on the results of a *K*-fold cross-validation. The whole dataset was randomly split into *K* = 10 approximately same-size folds. Each fold was kept apart for the validation and the remaining *K*−1 folds were used as a calibration dataset. Models were compared using the mean error (ME), the root mean square error (RMSE), the squared Pearson’s *r* correlation coefficient (*r*^2^), and the modelling efficiency coefficient (MEC), defined by the following equations:

Mean error:

1 $${\rm{ME}}=\frac{1}{n}\mathop{\sum }\limits_{i=1}^{n}{z}_{i}-{\widehat{z}}_{i},$$

where *z* and $\widehat{z}$ denote the measured and predicted values of TOC, respectively, and *n* is the total number of measured values. The ME represent the bias, i.e. the systematic over- or under-prediction of the model.

Root mean square error:

2 $${\rm{RMSE}}=\sqrt{\frac{1}{n}\mathop{\sum }\limits_{i=1}^{n}{({z}_{i}-{\widehat{z}}_{i})}^{2}}.$$

The RMSE represents the magnitude of the error, its optimal value is 0 and is expressed in the unit of TOC (i.e. in %).

Squared Pearson’s *r* correlation coefficient:

3 $${r}^{2}={\left(\frac{\mathop{\sum }\limits_{i=1}^{n}\left({z}_{i}-\overline{z}\right)\left({\widehat{z}}_{i}-\overline{\widehat{z}}\right)}{\sqrt{\mathop{\sum }\limits_{i=1}^{n}{\left({z}_{i}-\overline{z}\right)}^{2}}\sqrt{\mathop{\sum }\limits_{i=1}^{n}{\left({\widehat{z}}_{i}-\overline{\widehat{z}}\right)}^{2}}}\right)}^{2},$$

where $\overline{z}$ is the mean of the measured values and $\overline{\widehat{z}}$ is the mean of the predicted values. The *r*^2^ describes the linear correlation between measured and predicted values and ranges between 0 (no linear correlation) to 1 (perfect linear correlation).

Modelling efficiency coefficient:

4 $${\rm{MEC}}=1-\frac{\mathop{\sum }\limits_{i=1}^{n}{\left({z}_{i}-{\widehat{z}}_{i}\right)}^{2}}{\mathop{\sum }\limits_{i=1}^{n}{\left({z}_{i}-\overline{z}\right)}^{2}}.$$

The MEC optimal value is 1 but it can be negative if the mean of the measured values is a better predictor than the model. Positive MEC values can be interpreted as an amount of variance explained by the model.

We use a solar diagram^26^ to exploit the relationship between statistical indices and compare the maps. In a Cartesian coordinate system, the x-axis represents the ME (Eq. 1), and the y-axis the standard deviation of the error (SDE). The distance from the origin to any point in the diagram is expressed in terms of RMSE (Eq. 2), which allows to see the individual contribution of ME and SDE to the RMSE. The ME, SDE and RMSE are standardized by the standard deviation of the observation. The solar diagram further includes information on the correlation (Eq. 3) and MEC (Eq. 4). In a solar diagram, the closer the point to the origin of the diagram, the better the map. More information on the statistical representation of the validation statistics is provided in ref. ^26^.

##### Uncertainty quantification

We report the depth-specific lower (q_0.05_) and upper (q_0.95_) limits of the 90% prediction interval with two maps. Validation of the uncertainty estimates are obtained through a so-called accuracy plot. In an accuracy plot, the proportion of cross-validation observations contained in each *q* prediction interval is calculated. Ideally, the proportion of observations covered by a *q* interval is approximately equal to the value of *q*. If the proportion of observations in *q* is greater than *q*, it suggests that the uncertainty is under-estimated, whereas a substantially smaller proportion of observations compared to the nominal value of *q* suggests an under-estimation of the uncertainty. The process is repeated for all *q*, and the values of *q* are plotted against the actual proportion of values covered by *q* in a scattergram. Ideally, all values should be close to the 1:1 line, which would mean that the uncertainty is adequately estimated. Note that we evaluate uncertainty for all *q* against cross-validation observations, but report the maps of the 90% prediction intervals only.

## Data Records

### Prediction results

The depth-specific maps of TOC are distributed through the CSIRO Data Access Portal. Each product contains a map of TOC and its upper and lower prediction intervals. The products are available for grid size cells of 30 m × 30 m^27^ and 90 m × 90 m^28^ and for the size depth intervals: 0–5 cm, 5–15 cm, 15–30 cm, 30–60 cm, 60–100 cm and 100–200 cm. The unit of TOC is in percent mass (%). The projection system is WGS84 (EPSG:4326) and maps have extent: 112.54449°E - 153.38239°E; 9.59539°S - 43.38329°S for the 30 m and 112.99958°E - 153.99958°E; 10.0004°S - 44.00042°S for the 90 m. There are 36 maps in total: 6 depths, each with two maps of lower and upper intervals, for two spatial resolutions. An example of prediction along with the lower and upper intervals (0.05 and 0.95 percentiles) is shown in Fig. 6 for the 0–5 cm depth interval and the 90 m × 90 m spatial resolution. The maps of TOC prediction for all depth intervals and the two spatial resolutions are shown in Fig. 4. For the 90 m × 90 m resolution maps only, they can be used and accessed directly as Cloud Optimised GeoTIFF files through the TERN Data Store^28^.

## Technical Validation

### Prediction accuracy

**Fig. 2:**
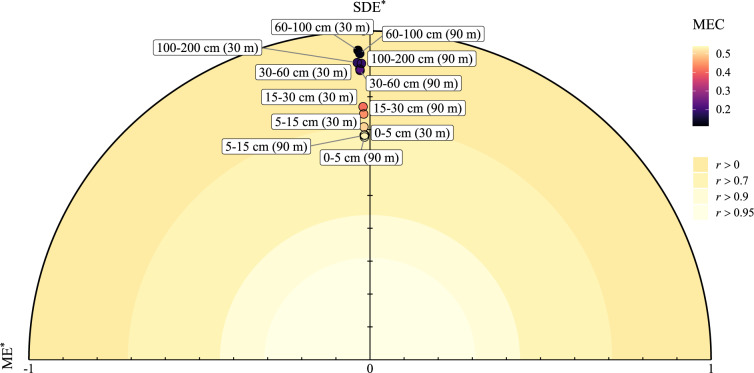
Summary diagram (solar diagram) of the validation statistics. The statistics are obtained by 10-fold cross-validation. The x-axis represent the mean error (ME), the y-axis is the standard deviation of the error (SDE) which are both standardized by the standard deviation of the observations (denoted by *). Any point in the diagram has a distance to the origin equal to the RMSE. Color scale indicate the Pearson’s *r* correlation coefficient and the modelling efficiency (MEC). Note that 30 m and 90 m refers to the map made at 30 m × 30 m and 90 m × 90 m resolutions, respectively.

The validation statistics for the QRF model prediction, two spatial resolutions and the six depth intervals are shown in Table 3 and visualized in Fig. 2. All maps have a negligible bias (the ME is close to 0 in all cases). The RMSE decreased with depth, which is expected because it is reported in the units of SOC (i.e. %) and the SOC content decreases with depth (see also Fig. 1). The solar diagram in Fig. 2 confirms that the RMSE is mostly composed of the SDE because all points are close to the a value of ME = 0. The *r*^2^ and MEC suggest that the models are accurate, but that prediction accuracy decreases dramatically for deeper depth intervals. This is an expected result reported in previous studies^14^. There is no substantial difference in terms of ME, *r*^2^ and MEC between the two spatial resolutions, and a slight improvement in RMSE between the prediction made with covariates at 30 m and that using 90 m resolution covariates.

**Table 3:** Depth-specific validation statistics for the maps.

	ME		RMSE		*r*^2^		MEC	
	30 m	90 m	30 m	90 m	30 m	90 m	30 m	90 m
0–5 m	0.03	0.03	1.25	1.39	0.53	0.53	0.53	0.53
5–15 cm	0.03	0.03	1.07	1.11	0.50	0.54	0.50	0.53
15–30 cm	0.02	0.02	0.90	0.93	0.41	0.44	0.41	0.44
30–60 cm	0.02	0.02	0.74	0.75	0.22	0.22	0.22	0.22
60–100 cm	0.02	0.02	0.50	0.56	0.12	0.13	0.11	0.12
100–200 cm	0.02	0.01	0.38	0.41	0.18	0.18	0.18	0.17

The statistics are obtained by 10-fold cross-validation. Note that 30 m and 90 m refers to the map made at 30 m × 30 m and 90 m × 90 m resolutions, respectively.

### Validation of the uncertainty

**Fig. 3:**
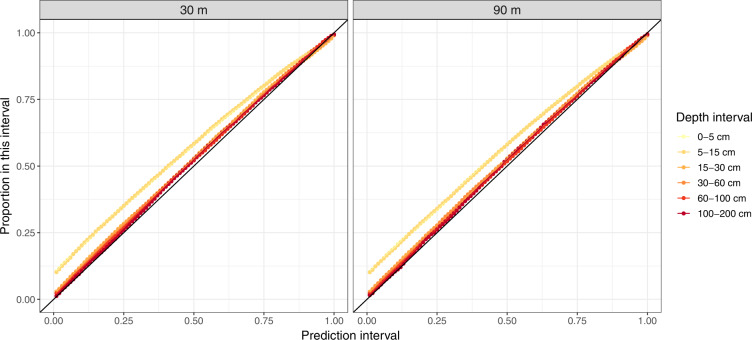
Accuracy plots showing the probability interval and the proportion falling within this interval, for the six depth intervals and two spatial resolutions.

**Fig. 4:**
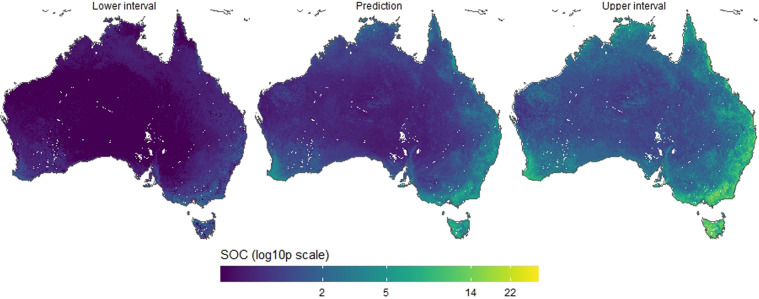
Map of prediction of TOC for the 0–5 cm depth interval and 90 m × 90 m spatial resolution (center) along with the lower (left) and upper (right) intervals (0.05 and 0.95 percentiles).

**Fig. 5:**
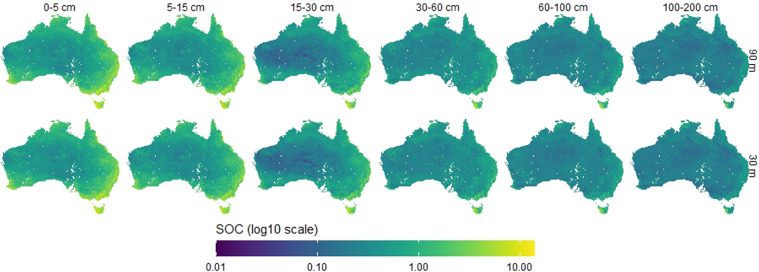
Maps of total organic carbon prediction for the size depth intervals and two spatial resolution (i.e. 90 m × 90 m and 30 m × 30 m).

The validation of the predicted prediction intervals is shown in Fig. 3. Note that while Fig. 2 shows all prediction intervals, we only report the maps of the 90% prediction interval, that is, the maps of the 0.05th and the 0.95 percentiles (see Figs. 4 and 5). Overall, the uncertainty was adequately estimated because most points are close to the line of equality. The uncertainty of SOC for the 0–5 cm depth intervals is slightly overestimated. For example, for the 50% interval, nearly 55% of the observations from the validation dataset fall within this interval.

### Comparison with existing maps

**Fig. 6:**
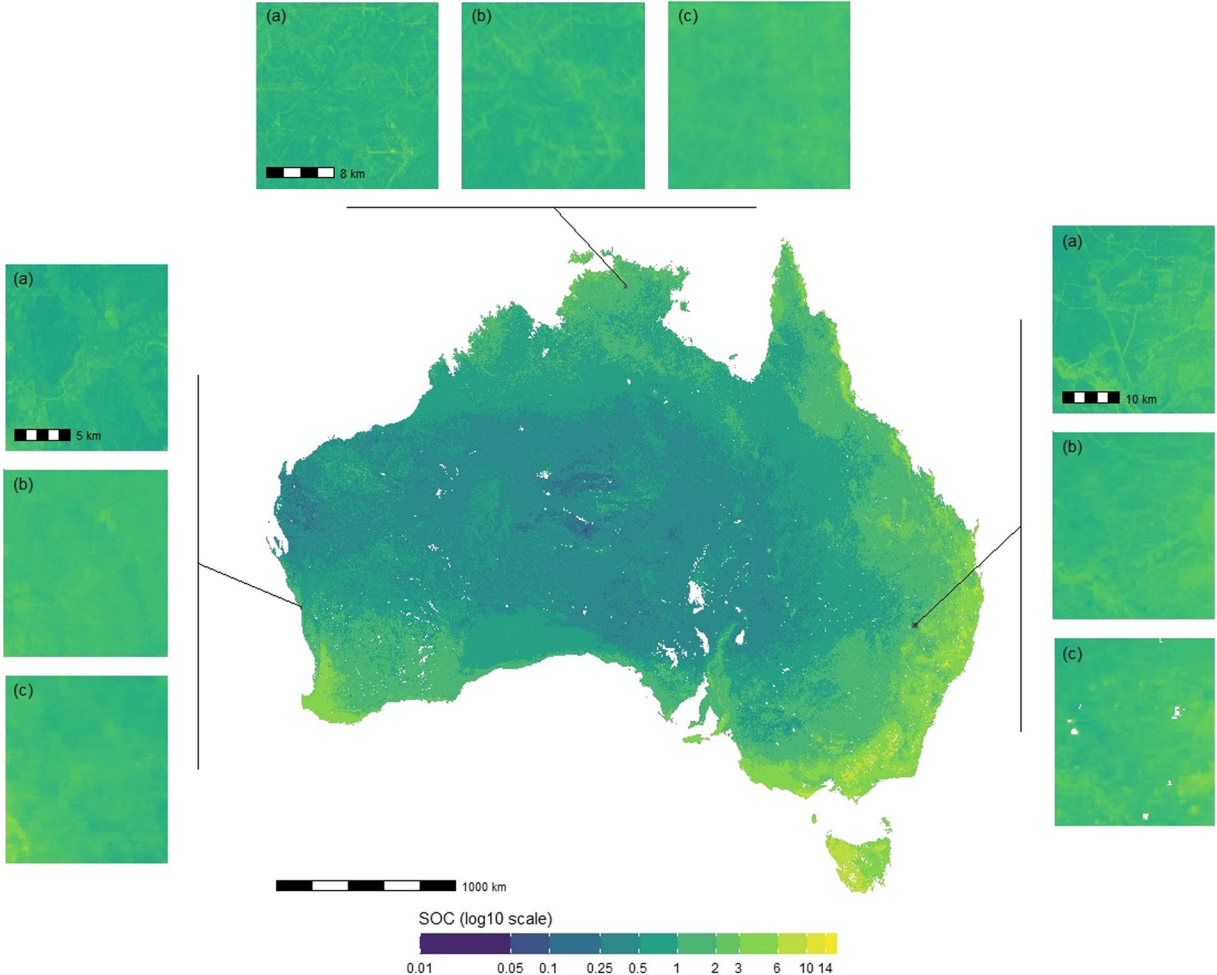
Topsoil (0–5 cm) (**a**) TOC prediction maps at 30 m × 30 m spatial resolution compared with (**b**) the previous version of the Soil and Landscape Grid of Australia and (**c**) the global map of SoilGrids 2.0^30^ for three small areas in the West, East and North of Australia.

We compared the topsoil (0–5 cm) TOC prediction maps at 30 m × 30 m (Fig. 6a) with existing maps available for Australia, the previous version of the SLGA v1 (Fig. 6b^29^) global map of SoilGrids 2.0 (Fig. 6c^30^). Note that in Fig. 6 the SLGA v1 map is at 90 m × 90 m whereas the SoilGrids map is at 250 m × 250 m spatial resolution. Three small areas in the West, East and North of Australia are shown. Overall, the prediction results for the three maps have similar patterns and ranges of values for the three small areas. However, the new map reveals much more detailed information than previous maps. The map of SoilGrids has smooth variation while the map of SLGV v1 captures further variation, but missed the detailed variation caused by fields and river beds.

## Usage Notes

With its high spatial resolution and national coverage, this dataset should be useful for a range of stakeholders, including policymakers, scientists and land users alike. Potential uses include setting up a benchmark for Australia to estimate the change in soil organic carbon resulting from a change in land use, land cover, soil management practices and greenhouse gas offset activities. They can be also used for researchers to obtain insights into the large-scale and local-scale drivers of soil organic carbon in relation to biophysical factors and the environment. National-scale maps of soil organic carbon also constitute an input to guide the design of national soil monitoring networks and soil organic carbon accounting projects. The uncertainty of the maps reported in this study can be used to guide sampling to refine the existing maps in areas of large uncertainty. At the international level, we envision that this map will help policymakers to report on the national soil carbon budgeting and might help assist with strategies to mitigate climate change through carbon storage in soils.

## Data Availability

Specific functions for data pre-processing and spline fitting are freely available in the R package ithir^31^. Codes associated to the model fitting, cross-validation and mapping are freely available from https://github.com/AusSoilsDSM/SLGA/tree/main/Production/DSM/SoilOrganicCarbon. All analyses were performed in in the R programming language (version 4.1.0).
